# Myofibroblast-like Cells and Junctional Complex Development Play a Role in Mouse Pubic Symphysis Remodeling During Pregnancy and Postpartum

**DOI:** 10.3390/ijms26115307

**Published:** 2025-05-31

**Authors:** Viviane Souza Rosa, Bianca Gazieri Castelucci, Monica Moreira, Paulo Pinto Joazeiro, Sílvio Roberto Consonni

**Affiliations:** 1 Department of Biochemistry and Tissue Biology, Institute of Biology (IB), State University of Campinas (Unicamp), Campinas 13083-970, Brazil; vdsrosa@mrc-lmb.cam.ac.uk (V.S.R.); bgcast@unicamp.br (B.G.C.); monica.moreira@upe.br (M.M.); pjoaz@unicamp.br (P.P.J.); 2 MRC Laboratory of Molecular Biology, Francis Crick Avenue, Trumpington, Cambridge CB2 0QH, UK; 3 Department of Genetic, Evolution, Microbiology and Immunology, Institute of Biology (IB), State University of Campinas (Unicamp), Campinas 13083-970, Brazil; 4 Oswaldo Cruz University Hospital (HUOC), University of Pernambuco (UPE), Recife 50100-130, Brazil

**Keywords:** pubic symphysis, pregnancy, myofibroblast-like cell, junctional complex

## Abstract

During mouse pregnancy, the pubic symphysis (PS) undergoes a gradual transitioning into an interpubic ligament (IpL) for a successful delivery. After birth, this IpL is rapidly remodeled, returning to the non-pregnant morphology. The PS fibrocartilaginous cells acquire a myofibroblast-like phenotype, characterized by extracellular matrix (ECM) secretion, expression of α-smooth muscle actin (α-SMA), and vimentin. While the presence of myofibroblast-like cells during the IpL remodeling is well described, cell–cell interactions and how this might contribute to the delivery remains poorly understood. This study uses ultrastructure and molecular approaches to investigate cell–cell and cell–ECM junctions during mouse pregnancy and postpartum. Our findings reveal that the intercellular contacts between adjacent IpL myofibroblast-like cells, particularly at late pregnancy stages, are characterized as adherens and GAP junctions. The acquisition of contractile elements by IpL cells, coupled with neighboring cells and the surrounding ECM via junctional complexes, suggests an important role in supporting changes in the mechanical forces generated by pubic bone movements during mouse pregnancy and also in tying the pelvic bones together, which may help the birth canal closure after delivery. Further studies in PS biology may investigate fibroblast to myofibroblast differentiation signaling cascades, which regulate the expression of pro-fibrotic proteins and may provide new insights for preterm labor.

## 1. Introduction

Mechanical forces transmitted through cell adhesion to the surrounding extracellular matrix (ECM), along with the dynamic rearrangement of cell–cell contacts and the cytoskeleton, have been shown to modulate changes in cell phenotype and function during development and physiological processes. These processes are crucial for tissue remodeling and homeostasis, highlighting the importance of bidirectional crosstalk between cells and their physical environment [1,2]. One fascinating example of mechanical forces inducing significant changes in ECM composition and phenotypic plasticity is observed in the mouse pubic symphysis (PS) remodeling during pregnancy, a structure that forms part of the birth canal, supports the pelvic organs, and allows the relaxation of the birth canal, ensuring a safe birth [3]. This mouse interpubic tissue remodeling is composed of different phases, separation, relaxation, and postpartum recovery [4,5], and occurs in others mammals, such as bats [6], guinea pigs [7], and, modestly, humans [8,9].

During mouse pregnancy, the fibrocartilaginous tissue, characteristic of the PS, gives rise to an interpubic ligament (IpL) during the separation phase from days 12 to 15 of pregnancy (D12-D15). The IpL is connected to the pubic bones through an osteoligamentous junction (OJ), or enthesis, a transitional tissue with a differential organization of cells and ECMs. By the end of pregnancy, days 18 and 19 (D18 and D19), the IpL and OJ cellular and ECM organization undergo drastic modifications, allowing the IpL to relax and extend, ensuring the separation of the pubic bones for safe delivery [10,11,12]. Then, after the first delivery, the IpL undergoes rapid involution, returning to its original size after about 10 days postpartum (dpp) [10,13,14,15,16]. These progressive modifications involve changes in the composition and rearrangement of ECMs, including collagen and elastic fibers [13,17,18], proteoglycans, glycosaminoglycans, and proteolytic enzymes mediating ECM degradation [4,19,20], as well as the proliferation and differentiation of fibroblasts and immune cells [4,18,20]. The change in the elastic extension of the mouse pubic joint showed a progressive increase from D12 to D19 and then an abrupt decrease 2–4 days postpartum [11], showing that the physical characteristics of collagen determine the flexibility and mechanical strength of the pubic joint. However, epidemiological studies have shown that many women fail to recover fully [21,22]. As pelvic organ support is related to the birth canal, its weakening may result in pelvic organ prolapse (POP) in women [23,24].

We previously showed that fibroblast-like cells in the IpL express cytoskeleton proteins such as vimentin and α-smooth muscle actin (α-SMA) and acquire abundant rough endoplasmic reticulum and Golgi complexes, which indicates that these have a role in the synthesis activity [12]. This behavior resembles that of myofibroblasts during wound healing [25,26]. Myofibroblasts were first identified as specialized contractile and ECM-secreting cells in granulation tissue of healing injuries, where they establish tension during contracture [25,27]. Fibroblasts begin to express α-SMA, the hallmark of myofibroblast differentiation, as well as myosin and vimentin under mechanical stress generated during tissue injury and repair and the presence of transforming growth factor β1 (TGF-β1) [18,28,29,30,31,32].

In the presence of mechanical stress, the cytoskeletal filaments converge into the myofibroblast surface, interacting with a complex of proteins, including talin, vinculin, and α-actinin, which is associated with transmembrane integrins and extracellular fibronectin, forming a cell–ECM adhesion complex known as a fibronexus [31,33,34,35]. This structure has been described as one of the key features of myofibroblast differentiation, and it is responsible for transmitting contractile force between the intracellular compartment and ECM and vice versa [36,37].

Myofibroblast differentiation has also been characterized by forming cell–cell adherens junctions by associating intracellular actin filaments with catenin proteins complexes and transmembrane cadherins [28,37,38]. Interestingly, a switch of cadherin expression occurs from N-cadherin in myofibroblasts of early wound granulation tissue to OB-cadherin in differentiated myofibroblasts, which is correlated with high levels of mechanical stress and stronger adhesion among these cells [39,40,41]. In addition to mechanical coupling, GAP junctions were demonstrated in myofibroblasts at wound granulation tissues in breast cancers and corneas [42,43,44,45]. GAP junctions are composed of hemichannels in the cell membrane that interconnect neighboring cells and allow the passage of small molecules. These channels are composed of transmembrane connexins, of which Connexin 43 seems to be the most common in fibroblasts and myofibroblasts [28,46].

The variety of cell–ECM and cell–cell junctions associated with the ECM synthesis features acquired during myofibroblast differentiation in wound contraction suggests that this cell may play an essential role in ECM remodeling in mouse PS during pregnancy because the interpubic tissue may form a multicellular contractile unit where mechanical and electrochemical cell coupling generate force, resulting in ECM reorganization [26]. However, the cell–ECM adhesion complex and the cell–cell adherens junctions have not been investigated in mouse PS remodeling during pregnancy and postpartum.

Although we previously showed that drastic changes in ECM and cell populations have been described during IpL formation and involution in the mouse PS throughout pregnancy and postpartum [4], it remains to be investigated whether this physiological (myo)fibroblast differentiation coincides with the establishment of junctional complexes during spatiotemporal IpL remodeling. Additionally, cervical ripening in both term and preterm labor correlates with changes in ECM components [47,48]. In pathological fibrosis, myofibroblasts lead to excessive ECM accumulation because TGF-β1 induces the expression of pro-fibrotic proteins [49]. Therefore, myofibroblast differentiation should be investigated to identify new insights into potential targets for understanding preterm labor. Here, considering elastic extension changes in a previous study [11], we describe myofibroblast differentiation at the IpL as marked by the acquisition of specialized cell–ECM and cell–cell junctions, which may be crucial for remodeling interpubic tissue at separation (D12-D15), relaxation (D15-D18), and postpartum recovery (3dpp) phases.

## 2. Results

### 2.1. Dynamic Changes on Cellular Phenotypes and Cell–Cell and Cell–ECM Interactions in the Interpubic Tissues During Pregnancy and Postpartum

**Figure 1:**
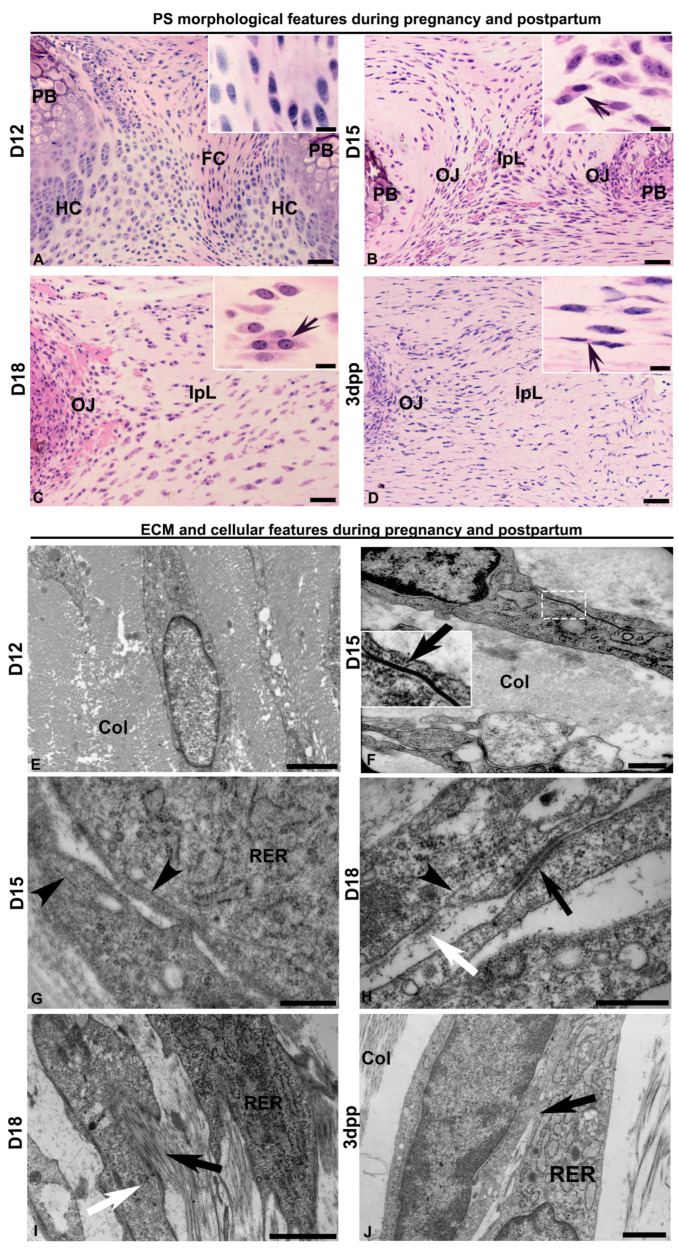
Cytoplasmic projections of the proximity morphology of fibroblast cells in a transversal section of the interpubic articulation during mouse pregnancy and postpartum. (**A**–**D**) Light and (**E**–**J**) electron micrographs of D12, D15, D18, and 3dpp. (**A**) Pubic symphysis (PS) is composed of fibrocartilage (FC) with fibrochondrocytes (detail) between hyaline cartilage (HC) continuous with the pubic bones (PBs). (**B**,**C**) The interpubic ligament (IpL) is continuous with the osteoligamentous junctions (OJs). (**D**) In the early postpartum days, the IpL starts to recover. (**B**–**D**) In detail, note the cytoplasmic proximity among cells (arrow). (**E**) Fibrochondrocytes show an elongated shape surrounded by collagen fibrils during early pregnancy at D12. (**F**) The gap junction between the cellular membranes of two adjacent fibroblast cells (arrow shown in the magnified white box detail). The cells are surrounded by densely packed collagen fibrils (Col) at D15. (**G**) Microtubules (arrowhead) close to the cell periphery in a two-cell contact at D15. (**H**) Microtubules (arrowhead) are close to electron-dense plaques between cellular membranes (black arrow) and are characteristic of adherent junctions. Note also the presence of a fibronexus (white arrow) at D18. (**I**) Projections of the plasma membrane are oriented in the same direction within the cytoskeleton fibers (white arrow) and surrounded by ECMs with thin collagen fibrils (black arrow) in their increased interfibrillar spaces at D18. (**J**) Fibroblast shows cytoplasm-rich in rough endoplasmic reticulum (RER) and cytoplasmic projection contacts (arrow) at the early postpartum stage. In panels (**A**–**D**): historesin sections stained with hematoxylin-phloxine B. Scale bars are 50 µm and, in details, 10 µm. (**E**–**J**): Transmission electron microscopy. Scale bars are 2 µm in panels (**E**,**J**), 1 µm in (**F**,**G**), and 500 nm in (**H**,**I**).

To characterize the dynamic changes in cellular phenotypes and ECM features that support the modifications of PS during pregnancy, we sought to examine the tissue and cell morphology of the interpubic tissues during pregnancy and postpartum. At D12, our results showed the fibrocartilaginous PS was composed mainly of elongated fibrochondrocytes at the central fibrocartilage between hyaline cartilage pads covering the pubic bone surfaces (Figure 1A). The replacement of fibrocartilaginous tissue by an IpL, a dense connective tissue, occurs during the separation phase from D12 to D15 (Figure 1B). Then, the IpL undergoes the relaxation phase with swift and pronounced swelling at D18 (Figure 1C). At this point, the IpL presents elongated fibroblast cells in a parallel position to the major axis of the joint, displaying cytoplasmic proximity among cells (insets in Figure 1B,C). At postpartum, the IpL undergoes rapid involution, but the cells still show a fibroblast-like phenotype with cytoplasmic proximity among cells (Figure 1D).

Ultrastructure reveals fibrochondrocytes immersed in the ECM which are composed of compacted thin collagen fibrils and organized in a dense network at D12 (Figure 1E). During the separation phase, fibroblasts show an elongated shape, cytoplasm rich in rough endoplasmic reticulum, a nucleus with loose chromatin, and the presence of cell–cell junctions in association with thicker collagen fibers (Figure 1F,G). During the relaxation phase, the ECM IpL comprises scattered, thin collagen fibrils with large spaces among them (Figure 1H,I). Interestingly, at D15 and D18, microtubules occur close to cytoplasmic projections among cells (Figure 1G,H), and the fibronexus is identified as an electron-dense region close to the cell membrane (Figure 1H).

We also observed a structure resembling the fibripositor, characterized by membrane cell projections associated with collagen fibrils that protrude from the cell’s main body and align with cytoskeleton fibers (Figure 1I). At 3dpp, fibroblasts still show cytoplasmic proximity among cells in the IpL (Figure 1J).

**Figure 2:**
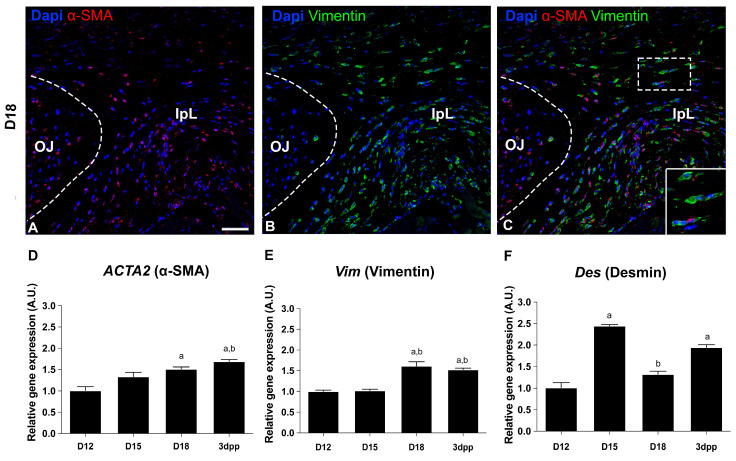
Analysis of myofibroblast profile during pregnancy and postpartum. (**A**–**C**) Immunolocalization of α-SMA (red) and vimentin (green) shows positive cells in the osteoligamentous junction (OJ) and the interpubic ligament (IpL) at D18. ((**C**)—in detail) IpL shows elongated cells double-positive for α-SMA and vimentin. Blue: DAPI. Scale bar: 50 µm. (**D**–**F**) Quantitative real-time PCR analysis of myofibroblast markers *ACTA2* (α-SMA), *Vim* (Vimentin), and *Des* (Desmin) during pregnancy and postpartum. Statistical analysis was performed by one-way ANOVA with Tukey’s multiple comparison test. N = three independent experiments. The data are presented as mean ± SEM, with significance at *p*-value < 0.05 indicated by: a vs. D12 and b vs. D15. A.U. = arbitrary unit.

To assess the phenotypic modulation of the cellular compartment from D15 to D18 in mouse PS, we investigate the presence of myofibroblast biomarkers by immunofluorescence and real-time PCR. In agreement with previous work [18], our results at D18 indicate that approximately 50% of the IpL cells display double-positive staining for α-SMA and vimentin, two major biomarkers of myofibroblasts. In contrast, in OJ cells, this percentage is about 10% (Figure 2A–C). Additionally, quantitative gene expression analysis showed a significant increase in α-*SMA* and *Vimentin* transcripts by the end of pregnancy and early postpartum compared to the interpubic tissues at D12 (Figure 2D,E). In contrast, *Desmin* expression is upregulated at D15 and 3dpp, and downregulated at D18 compared to D12 (Figure 2F). Taken together, our data provide strong evidence that the cells from IpL acquire myofibroblast-like features during late pregnancy and early postpartum in mice.

### 2.2. Cell–Cell and Cell–ECM Junctions Play a Role in the PS Remodeling During Pregnancy and Postpartum

Through our morphological and ultrastructural analysis, we found evidence of cell–cell and cell–ECM interactions. We then performed a detailed spatiotemporal study of connexin 43 and β-catenin expression to analyze the presence of GAP junctions and adherens junctions, respectively, using immunofluorescence in the previously identified myofibroblast-like cells.

**Figure 3:**
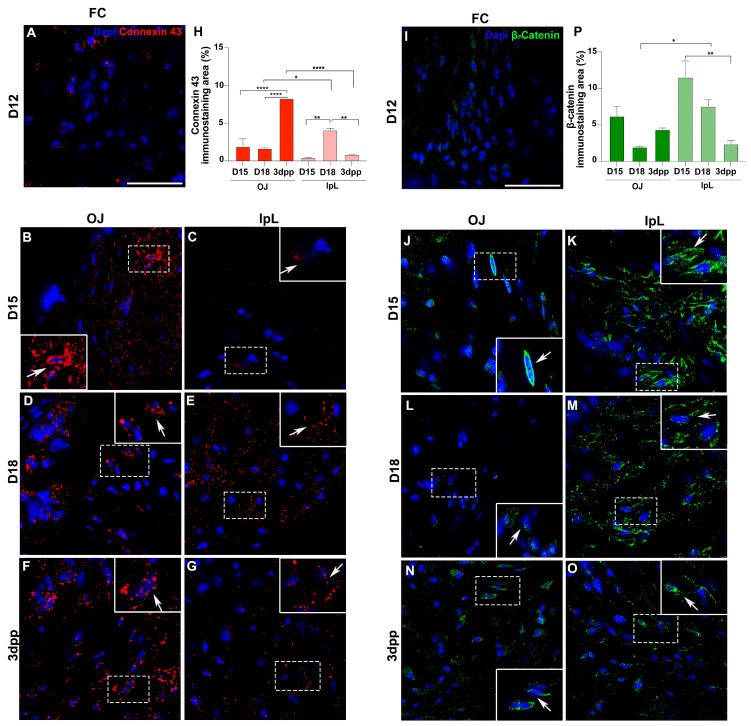
Immunolocalization of intercellular junctions during pregnancy and postpartum in the PS and IpL. Immunolocalization of connexin 43 (red) and β-catenin (green) in fibrocartilage (FC) of the pubic symphysis at D12 (**A**,**I**), and in the osteoligamentous junctions (OJs) or the interpubic ligament (IpL) at D15 (**B**,**C**,**J**,**K**), D18 (**D**,**E**,**L**,**M**), and 3dpp (**F**,**G**,**N**,**O**). In detail, note the localization of connexin 43 and β-catenin preferentially around the nucleus (arrows) of cells in the OJ and the cell cytoplasm (arrows) in the IpL. Blue: DAPI. Scale bars: 50 µm. Percentage of connexin 43 and β-catenin immunostaining area (**H**,**P**). Statistical analysis was performed by one-way ANOVA with Tukey’s multiple comparison test. N = three samples per group. The data are presented as mean ± SEM, with significance indicated by a *p*-value <0.05 (*), <0.01 (**), <0.0001 (****).

For connexin 43, rare positive cells are seen in the FC at D12 (Figure 3A). At D15, cells show intense staining for connexin 43, mainly at the OJ (Figure 3B,C,H). At D18, positive cells for connexin 43 are seen at both the OJ and IpL, with a larger immunostaining area observed in the IpL (Figure 3D,E,H). At postpartum, positive cells for connexin 43 are found at the OJ and IpL (Figure 3F,G,H). Notably, while 3dpp shows the highest immunostaining area in the OJ, on the IpL region the largest connexin 43 staining area is observed at D18 (Figure 3H).

**Figure 4:**
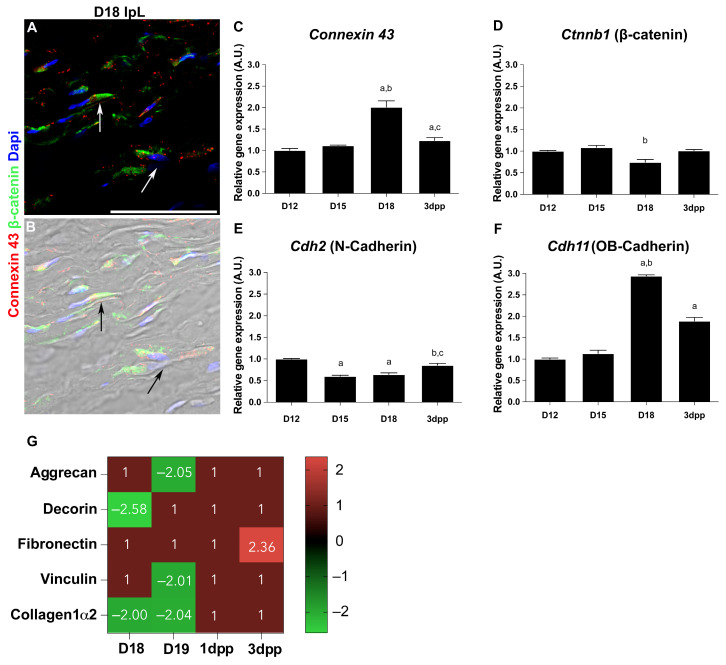
Analysis of cell–cell and cell–ECM interactions during interpubic tissue remodeling. (**A**,**B**) Double-staining of connexin 43 (red) and β-catenin (green) showing both junctional proteins in the cytoplasm of cells in the relaxed D18 IpL (white and black arrows indicate the presence of connexin 43 and β-catenin in the cytoplasm projections of two adjacent cells). (**B**) Brightfield image from (**A**). Quantitative real-time PCR analysis of *Connexin 43* (**C**), ***Ctnnb1*** (β-catenin) (**D**), ***Cdh2*** (N-Cadherin) (**E**), and ***Cdh11*** (OB-Cadherin) (**F**) at D12, D15, D18, and 3dpp. Statistical analysis was performed by one-way ANOVA with Tukey’s multiple comparison test. The data are presented as mean ± SEM with significance (*p* < 0.05) indicated by a vs. D12, b vs. D15, and c vs. D18. N = three independent experiments. A.U. = arbitrary unit. (**G**) Quantitative data of protein expression levels of aggrecan, decorin, fibronectin, vinculin, and collagen1α2 at the interpubic tissues from D18 to 3dpp. The data were obtained by shotgun proteomic analysis, with a *p*-value < 0.05 indicating statistical significance. The differentially expressed proteins were obtained after normalization to D12 levels. The complete data can be accessed in [20].

For β-catenin, rare positive cells are observed in the FC at D12 (Figure 3I). However, immunolocalization reveals positive cells in both the OJ and IpL from D15 to 3dpp (Figure 3J–P), particularly during IpL formation and relaxation (Figure 3K,M,P). The expression pattern is different according to their subcellular localization. Whereas OJ cells show β-catenin in the perinuclear area, IpL cells display β-catenin spread through the cytoplasm (Figure 3J–O). Additionally, we found double-positive cells for β-catenin and connexin 43 in the IpL at D18 (Figure 4A,B).

To shed light on the morphological evidence, the gene expression of *Connexin 43* for GAP junctions and *Ctnnb1* (β-catenin) for adherens junctions was assessed by qPCR. Additionally, we investigated a possible shift in gene expression between Cdh2 (N-cadherin) and Cdh11 (OB-cadherin) as they have been recognized as differentiation markers from a fibroblast to a myofibroblast [40,41]. Our results show that *Connexin 43* and *Cdh11* (OB-cadherin) levels increased significantly at D18 and 3dpp compared to D12 (Figure 4C,F). Interestingly, the opposite is observed in *Ctnnb1* (β-catenin), where gene expression decreases only at D18 (Figure 4D), and for Cdh2 (N-cadherin), which decreases at D15 and D18 (Figure 4E). At postpartum, in both *Ctnnb1* and *Cdh2*, the expression levels are restored to D12 levels (Figure 4D,E).

To gain further insights into how ECM remodeling is temporally associated with the acquisition of cell–cell and cell–ECM junctions during myofibroblast differentiation, we sought to refine specifically the quantitative data on protein levels of aggrecan, decorin, fibronectin, vinculin, and collagen1α2 from a previous proteomic analysis in the PS [20]. The in silico analysis demonstrates that collagen1α2 protein levels decrease at D18 and D19, when the IpL is entirely relaxed, but return to similar levels to those at D12 by early postpartum days (Figure 4G). Comparable decreased protein levels are observed for the proteoglycans aggrecan at D19 and decorin at D18 (Figure 4G). Next, we focused on the fibronexus elements vinculin and fibronectin [26], where vinculin protein levels decrease at D19, while fibronectin levels remain stable when the IpL is relaxed and increase at 3dpp (Figure 4G).

## 3. Discussion

During mouse pregnancy and delivery, the plasticity of cell populations and ECM components are crucial for maintaining interpubic tissue homeostasis to ensure a safe passage of the fetus through the birth canal [4,13,14,15,17,18]. This remodeling during the first pregnancy can be observed by comparing the average width of the interpubic gap at D12, which is approximately 0.3 mm. The data for the average width on other days is as follows: D15, 1.1 mm; D19, 5.0 mm; and 3dpp, 1.4 mm [50]. Our study provides new insights suggesting that the success of interpubic tissue remodeling may depend on mechanotransduction signals between the ECM and cellular compartments. This finding is demonstrated by the dynamic differentiation of myofibroblast-like cells through the development of cell–cell and cell–ECM junctions. Myofibroblast-like cells exhibit contractile features and interact with each other and with the ECM through the junction complex [28,40], possibly playing an active and important role in the IpL relaxation during pregnancy and recovery at postpartum, tying the pelvic bones together, which may facilitate the closure of the birth canal after delivery.

Our analysis enabled us to evaluate the classical IpL formation, relaxation, and involution events during the first pregnancy of mice [6,11,12,13,18]. Since the IpL relaxation process is precisely regulated by estrogen, progesterone, and relaxin, the presence of high levels of their receptors in the fibrochondrocytes of the PS of virgin female mice might indicate that these target cells respond to the stimuli for cell proliferation, apoptosis, differentiation, and ECM turnover [2]. Without altering the proportions of collagen types, relaxin has been shown to decrease total collagen content, which alters collagen metabolism in the interpubic ligament and cervix of mice and rats [51,52]. The IpL and cervix have been shown not to grow and relax during pregnancy in relaxin-deficient mice as this deficiency disrupts collagen breakdown and reorganization, which may contribute to a parturition defect in some of these animals [53,54].

Specifically, the dynamic differentiation process occurs during the IpL relaxation as fibroblasts acquire an elongated shape and cytoplasmic projections, organize actin and microtubule filaments at the cell periphery, and increase their cytoplasmic organelles such as RER. Additionally, higher expression of cytoskeleton markers, such as α-SMA and vimentin, together with the downregulation of desmin, which is not expressed by myofibroblast [31,32] at later stages of pregnancy, supports the hypothesis that fibroblast cells acquire synthesis features and specialized contractile apparatus similar to those displayed by myofibroblasts [26,36].

Myofibroblast differentiation is coordinately regulated by a mechanically stressed environment, which induces α-SMA expression, conferring the ability to contract to these cells and playing a key role during tissue remodeling [26,30]. Our results demonstrated that fibroblasts from IpL acquire contractile features, as evidenced by the ultrastructure, gene, and protein expression analysis. The acquisition of contractile machinery by these cells may significantly contribute to IpL relaxation, supporting mechanical force changes in the pelvic region [18]. Likewise, myofibroblasts are described as participants in synthesizing ECM compounds, such as collagen and fibronectin, and are responsible for producing enzymes involved in matrix degradation [31,55]. Both processes have been extensively characterized at PS during pregnancy and are essential for IpL formation and involution at postpartum [2,4,13,19,56]. In pathological fibrosis, myofibroblasts cause excessive ECM accumulation because TGF-β1, through Smad2/3 signaling, induces the expression of pro-fibrotic proteins [49,57,58].

In humans, massive ECM remodeling occurs during pregnancy and birth, and a condition that has implications in urogynecological practice is POP which is considered a high risk for preterm labor [59]. Despite the differences between mice and humans [60,61,62], genetically modified mice for enzymes responsible for elastin fiber assembly [63,64,65,66] have been used for a wide variety of investigations, including genetic pathways involved in the development of POP and biomechanical properties of the pelvic floor as these animal models develop POP similar to humans. Cervical ripening in term and preterm labor correlates with changes in ECM components [47,48], and myofibroblast differentiation prevents excessive reduction in cervical collagen content and inhibits premature cervical ripening [67]. Therefore, it is imperative to investigate PS myofibroblast signaling cascade differentiation to provide new insights into potential targets for understanding preterm labor.

Specifically, in terms of collagen reorganization, there is a transition of densely packed fibers at early stages of pregnancy to sparsely thin fibrils with large spaces filled by proteoglycans and glycosaminoglycans when the IpL displays a high level of relaxation [4,17,18,19]. The mechanism of collagen fiber reorganization after pregnancy remains unclear; however, the presence of fibripositor-like structures, responsible for the deposition of new collagen fibrils in tendons during embryonic development [68,69], observed in the IpL at later stages, along with a decrease in collagen1α2 protein levels at the end of pregnancy, suggests that the collagen reorganization at the PS might involve both the degradation and synthesis of new collagen fibers.

Although fibripositor formation has traditionally been described only during a narrow window of embryonic development when tissue architecture is being established [68], the biomechanical changes at PS during pregnancy and postpartum, along with our data, indicate that the fibripositors might be present in late pregnancy at the PS, likely playing a role during ECM remodeling.

Interactions between ECM proteins and the cytoskeleton are crucial for transmitting force and signaling between intracellular and extracellular compartments, providing cell–cell communication through stress fields in the surrounding ECM [37]. The presence of α-SMA and microtubules at the cell periphery has also been described in many other cell types as a trigger for cell–cell junctions and fibronexus assembly, which is a fundamental complex that facilitates migration, mechanotransduction, and supports the formation and stability of cell–cell and cell–ECM interactions [37,40,70,71,72]. During pubic bone displacement, fibronexus may play a role in cell–ECM mechanotransduction signals, inducing morphological and functional changes in the cell population. Myofibroblast-like cells and fibronexuses have been previously described in the uterine cervix [73] and PS during mouse pregnancy [18]. However, to our knowledge, there are still no data correlating myofibroblasts with the presence and dynamics of cellular junctions’ development during pregnancy and postpartum at the PS.

According to our ultrastructural and molecular experiments, the cytoplasmic projections’ proximity observed among parallel cells mainly at D15 and D18 corresponds to GAP and adherent junctions, previously described during myofibroblast differentiation in granulation tissue [37]. Interestingly, the expression of connexin 43 and β-catenin seemed to be spatiotemporally regulated at the OJ and IpL during pregnancy and postpartum. The OJ comprises various tissues and is critical for transmitting forces applied to pubic bones [10,16,74]. In other systems, such as the tendon, mechanical forces are necessary for both developing and maintaining the enthesis. Furthermore, recent reports showed how the enthesis cellular compartment can respond to mechanical changes in a physiological context [75]. Thus, the differential expression of connexin 43 and β-catenin observed in cells at the OJ and IpL supports the hypothesis that distinct roles, regulation, and mechanical forces occur in each area during IpL formation, relaxation, and recovery [10,16].

Although there is no clear consensus on whether connexin 43 is a specific marker of myofibroblast differentiation, since it also regulates the communication between fibroblasts [28], our results have undoubtedly shown an upregulation of connexin 43 at late stages of pregnancy at the interpubic articulation. Upregulation of connexin 43 is also described in late pregnancy in the myometrium, which is essential for developing uterine contractions during labor in mice, and the specific deletion of connexin 43 in uterine smooth muscle cells results in prolonged pregnancy and a subsequent delay in delivery [76].

On the other hand, the specificity of the adherens junction during myofibroblast differentiation is widely demonstrated [28,40]. In our study, we demonstrated that β-catenin is immunolocalized in the cytoplasm of cells in the IpL from D15 to 3dpp, despite the downregulation of its gene expression at D18. These findings agree with previous studies, which reported a decrease in α-, β-catenin, and N-cadherin expression during myofibroblast differentiation. Precisely, the downregulation of α- and β-catenin reflects a reduction in the number of cell–cell contacts and, consequently, the establishment of smaller aggregates of myofibroblasts, which contributes to a balance of force generation and its transmission to the ECM [40].

Besides β-catenin expression, our analysis has shown distinguished expression patterns for N-cadherin and OB-cadherin. While OB-cadherin gene expression is upregulated at the time of IpL formation and relaxation, N-cadherin is downregulated. In vitro and in vivo experiments focusing on myofibroblast differentiation have shown that this shift between N and OB-cadherin is correlated with high levels of mechanical stress and stronger adhesion among cells, providing high mechanical resistance to extracellularly applied forces and improving cellular force generation [39,40,41]. Then, the cadherin’s expression pattern strengthens the hypothesis that IpL relaxation and involution are marked by differentiation from fibroblast to myofibroblast, which is a remarkable example of cellular plasticity to adapt junctional complexes due to the mechanical environment and ECM remodeling in the PS during pregnancy and postpartum.

This variety and spatiotemporal patterning regulation of cellular junctions establish a contractile and mechanically resistant multicellular unit, which might operate in the remodeling of interpubic tissue and support the variations in mechanical forces caused by pubic bone displacement: this is similar to that described in granulation tissues during wound healing [26,28,39,40].

Cell–cell and cell–ECM interactions are crucial for maintaining homeostasis and facilitating tissue remodeling. Recent studies have shown a complementary correlation between cell and ECM junctions, where the assembly of a fibronectin matrix with transmembrane integrins leads to the disruption of cadherin-mediated cell–cell adhesion [77,78]. Although our study did not specifically focus on the correlation between cell–matrix adhesion and cell–cell junctions at the interpubic articulation, our analysis of proteomic data from our study group [20], along with our gene expression analysis, showed downregulation of fibronexus markers at later pregnancy. In contrast, the cell–cell junction markers, connexin 43 and OB-cadherin, were upregulated, suggesting the predominance of cell–cell junctions in the IpL. Future functional experiments involving the inhibition or overexpression of junctional complex proteins at the mouse IpL would better clarify their role in myofibroblast differentiation and interpubic tissue relaxation.

At postpartum, when the pubic bones reapproximate, the gene expression level of connexin 43 and OB-cadherin decreased from 1dpp to 3dpp. In contrast, the protein levels of fibronectin and vinculin were upregulated, highlighting the critical role of the fibronexus in mechanotransduction response due to the return of the pubic bones. Moreover, the increase in cell–cell junctions and decrease in cell–ECM interactions at the interpubic tissues occur when the IpL shows the highest level of relaxation, which is at the end of pregnancy. This period is characterized by the rearrangement of collagen and elastic fibers along with changes in the composition of proteoglycans and hyaluronic acid [4,19,20], displaying lower levels of collagen type Iα2 and proteoglycans, such as aggrecan and decorin, which are upregulated shortly after parturition when the fibrocartilaginous tissue is restored.

Current insights have advanced our understanding of this essential facet of the mechanical forces during the IpL formation, relaxation, and recovery, which involve mechanotransduction from the ECM to the fibroblast. Although nerve distribution or blood supply were not our core research in this work, for a more comprehensive understanding of the overall mouse tissue remodeling process it is important to consider that most of the muscle fibers inserted into or near the ligament are displaced outwards with the separating pubic bones [11]. Despite the avascular cartilage matrix, an adequate blood supply is present around the PS [9]. However, for urogynecological studies and clinical practices, it is imperative to consider the complexity of nerve distribution and blood supply during PS remodeling [79].

## 4. Materials and Methods

### 4.1. Animals

Virgin female C57BL/6/JUnib mice (3 months old) were obtained from the Multidisciplinary Center for Biological Investigation on Laboratory Animal Science (CEMIB) at UNICAMP. Mating was encouraged by placing the young females in the same cage with breeding males overnight. Vaginal plug formation was considered to be an indicator of the day 1 of pregnancy (D1).

PSs or IpLs were obtained from the following groups: day 12 of pregnancy (D12), D15, D18, and 3 days postpartum (3dpp). The animals were anesthetized using a mixture of 100–200 mg/kg ketamine and 5–16 mg/kg xylazine chloride (Agribrands do Brasil, Jacarei, Brazil), which was administered intraperitoneally between 11:00 a.m. and 12:00 p.m. The mice were sacrificed by cervical dislocation, and the symphyses or ligaments were then removed and processed. Considering the statistical power of 0.90 and the significance criterion of 0.05, according to [80], we used three animals per group for light microscopy, immunofluorescence, and transmission electron microscopy. Three (D18) or six (D12, D15, and 3dpp) animals were used for real-time PCR analysis to have sufficient tissue for satisfactory total RNA extraction. A total of 57 animals were used for the experimental studies. The animal experiments were conducted by the Guide for the Care and Use of Laboratory Animals, issued by the National Institutes of Health (NIH; Bethesda, MD, USA). All experimental protocols were approved by the Institutional Committee for Ethics in Animal Experimentation (CEEA/IB/UNICAMP), protocol n° 2325-1).

### 4.2. Histology

The PS and IpL were removed, fixed, processed, and embedded in historesin (Leica Microsystems, Heidelberg, Germany) as described previously [81] and sectioned transversely (in the anteroposterior direction) at a width of 3 μm. According to Bennett and coworkers [82], the sections were mounted on slides and stained with hematoxylin-phloxine B (Sigma). After staining, the samples were imaged using a Nikon Eclipse E800 light microscope (Nikon Corporation, Tokyo, Japan).

### 4.3. Transmission Electron Microscopy

Small samples of PS and IpL from three animals per group of study (D12, D15, D18, and 3dpp) were fixed in 2.5% glutaraldehyde (Electron Microscope Science, Hatfield, PA, USA) in 0.1 M cacodylate buffer containing 0.3% tannic acid for 4 h at 4 °C. The tissues were post-fixed with 1% osmium in 0.1 M cacodylate buffer for 1 h at 4 °C, dehydrated through a graded series of acetone, and embedded in Epon resin (EMbed-812; Electron Microscopy Sciences, Hatfield, PA, USA). Ultrathin sections were stained with uranyl acetate and lead citrate. The sections were examined using a LEO 906 electron microscope (Electron Microscopy Laboratory, IB, UNICAMP, Campinas, Brazil).

### 4.4. Immunofluorescence 

Immunofluorescence was used to determine the cellular locations of connexin 43, β-catenin, α-SMA, and vimentin. Staining was performed on cryosections (8 μm) of PS and IpL, which were frozen in n-hexane with liquid nitrogen. The cryosections were fixed with acetone at −20 °C for 3 min, and then the slides were washed in 0.1 M PBS (pH 7.4). After blocking with PBS and 1% bovine serum albumin (BSA) for 30 min, the sections were incubated with a primary antibody diluted at 1:100 in PBS with 1% BSA at 4 °C overnight. The following were used, connexin 43 (Invitrogen, 71-0700), β-catenin (Santa Cruz, sc-1496), α-SMA (Biocare Medical, Concord, CA, USA, CM 001 A, B, C), and vimentin (Abcam, Cambridge, UK, ab45939). 

The incubation with the primary antibodies was followed by incubation with the respective secondary antibodies diluted in PBS with 1% BSA. The secondary antibodies used were Donkey anti-Goat AlexaFluor488 (ThermoFisher, Waltham, MA, USA, A-11055), Donkey anti-Rabbit AlexaFluor555 (ThermoFisher, A-31572), Donkey anti-Rabbit AlexaFluor488 (ThermoFisher, A-21206), and Donkey anti-Mouse AlexaFluor555 (ThermoFisher, A-31570). The nuclei were stained using 4′,6-diamidino-2-fenilindol (DAPI, SC-3598, Santa Cruz). The sections were mounted with Vectashield Mounting Medium (Vector Labs, Burlingame, CA, USA). The images were acquired on an inverted Confocal Zeiss LSM 780-NLO microscope (National Institute of Science and Technology on Photonics Applied to Cell Biology-INFABIC, UNICAMP, Campinas, Brazil) or a Zeiss LSM-510 (Hematology and Hemotherapy Center, Faculty of Medical Sciences, UNICAMP, Campinas, Brazil) using the 20× and 40× objectives. The images were processed using Fiji software, version 2.9.0 [83].

### 4.5. Quantification of Immunofluorescence Images

Vimentin and α-SMA double-positive cells were determined by first creating a DAPI mask and using the cell counter plugin in Fiji [84] to count the total number of cells in an area, and then manually counting all double-positive cells for both vimentin and α-SMA. Three random images were analyzed from each animal for each day of pregnancy and postpartum.

Connexin 43 and β-catenin channels were used to create a mask for the analysis of the immunostaining areas. Three random images were analyzed from each animal for each day of pregnancy and postpartum to determine the total and the percentage of immunostaining area using the “Analyse particles” function in Fiji [84].

### 4.6. Real-Time PCR

Gene expression was assessed by quantitative real-time PCR in the D12, D15, D18, and 3dpp groups. Total RNA was extracted from frozen tissues using TRIzol Reagent (Invitrogen, Carlsbad, CA, USA), and cDNA was synthesized using a RevertAid H Minus First Strand cDNA Synthesis Kit (Fermentas, Glen Burnie, MD, USA). Both procedures were carried out according to the manufacturer’s recommendations. Real-time PCR was performed using SYBR Green (Applied Biosystems, Foster City, CA, USA) in an Applied Biosystems 7300. Each gene was normalized to the expression of the housekeeping gene *36b4,* officially known as ribosomal protein, large, P0 (*Rplp0*), an estradiol-independent mRNA control [85]. The primers for *Connexin 43*, ***Ctnnb1*** (β-catenin), ***Cdh2*** (N-Cadherin), ***Cdh11*** (OB-Cadherin), *ACTA2* (α-SMA), *Vim* (Vimentin), and *Des* (Desmin) were purchased from IDT (Integrated DNA Technologies, Coralville, IA, USA), and the sequences are shown in Supplemental Table S1. All primers were optimized, and dissociation curves were prepared to ensure that only one product was amplified. A total of 20 ng of cDNA was used in each reaction according to the universal cycling conditions for the SYBR Green system.

The results were normalized using the CT (threshold cycle) values of the housekeeping gene *36b4*. To quantify and acquire the fold increase in the genes, the mathematical model 2^−ΔΔCt^ was utilized, normalizing to the D12 group because this PS histoarchitecture is quite similar to that of a nonpregnant mouse [17]. However, the tissues are subjected to hormonal regulation during pregnancy. Three (D18) or six (D12, D15, and 3dpp) animals were used for each experimental time point, and all reactions were performed in triplicate on the same plate.

### 4.7. Proteomic Shotgun Analysis

To gain further insights into how ECM remodeling is temporally associated with the acquisition of cell–cell and cell–ECM junctions during myofibroblast differentiation, we sought to refine specifically quantitative data of protein levels of aggrecan, decorin, fibronectin, vinculin, and collagen1α2 from a previous proteomic analysis in the PS [20]. Briefly, PS and IpL proteins were extracted using a PlusOne Sample Grinding Kit (GE Healthcare, Little Chalfont, Bucks, UK), according to the manufacturer’s protocol. For fractionation and elution, we have used a 2D ACQUITY UPLC M-Class system (Waters Corporation, Manchester, UK) coupled to a SYNAPT G2-Si Mass Spectrometer (Waters Corp.) and an XBridge BEH130 C18:5 µm × 300 µm × 50 mm column (Waters Corp.). Peptides were separated on an HSS T3: 1.8 µm × 75 µm × 150 mm column (Waters Corp.), and mass spectrometry was performed using nanoESI ionization in positive mode and data acquisition using HDMSE (Waters Corp.). Progenesis QI for Proteomics v3.0 software (Waters Corp.) was used for protein identification and quantitation.

The protein search was performed against the mouse revised databank from UniProt, using the search parameters detailed by [86]. After identification, DEPs were processed using one quick analysis tool in the MetaCore database (Clarivate Analytics), and only processes in which DEPs were grouped with a false discovery rate (FDR) < 0.01 were selected for analysis.

### 4.8. Statistical Analysis

Statistical analyses for immunofluorescence data and real-time PCR were performed using GraphPad Prism 10.5.0 (GraphPad Software, Inc., La Jolla, CA, USA). The relative gene expression analysis was performed using rank data classification and semiparametric analysis [87,88]. We also used a one-way ANOVA followed by Tukey’s test, with *p* < 0.05 indicating significance.

Three images from each group were used to quantify the immunostaining area of connexin 43 and β-catenin. The normality of the data was confirmed using a Shapiro–Wilk test. Then, an ANOVA test was followed by Tukey’s multiple comparisons test.

All data are presented as the mean values ± standard errors of the mean (SEM).

## 5. Conclusions

Our findings demonstrate that the mechanical forces during the IpL formation, relaxation, and recovery involve mechanotransduction from the ECM to fibroblasts through the fibronexus, which in turn induces morphological and functional changes in these cells, leading them to acquire myofibroblast features. Myofibroblast-like cells may act as contractile and mechanically resistant elements, establishing intercellular junctions, particularly at the end of pregnancy. These changes likely play a crucial role in the remodeling of interpubic tissues, ensuring tissue homeostasis and mechanical stability during IpL relaxation in pregnancy and IpL recovery at first postpartum, tying the pelvic bones together. Further studies in PS biology may investigate fibroblast to myofibroblast differentiation signaling cascades, which regulate the expression of pro-fibrotic proteins and may provide new insights into potential targets for understanding preterm labor.

## Data Availability

The original contributions presented in the study are included in the article/supplementary material, further inquiries can be directed to the corresponding author.

## Supplementary Materials

The following supporting information can be downloaded at: https://www.mdpi.com/article/10.3390/ijms26115307/s1.
