# Supplementary material for: Myofibroblast-like Cells and Junctional Complex Development Play a Role in Mouse Pubic Symphysis Remodeling During Pregnancy and Postpartum
Source: Int J Mol Sci. 2025 May 31;26(11):5307. doi: 10.3390/ijms26115307 (PMC12154126; doi:10.3390/ijms26115307)
Supplement: Supplementary file 1 [file ijms-26-05307-s001.zip › ijms-3560599-supplementary.pdf]

Supplemental table S1 – Primers Sequences

Table S1: List of primers used in the qPCR analysis.

Gene	Sequence	Amplicon
Connexin 43	F:TGGACAAGGTCCAAGCCTAC<br>R: ACAGCGAAAGGCAGACTGTT	131
Ctnnb1 (β-catenin)	F:CGCGTCAGCTCGTGTCCTGTG<br>R:CTTCAGGTACCCTCAGGCCCGC	141
Cdh2 (N-Cadherin)	F: GCCAACCTAACTGTCACGGA<br>R: GGGTCTGTCAGGATGGCAAA	110
Cdh11 (OB-Cadherin)	F:ACTTGTGAATGGGACTCGGAC<br>R: ACAGTAATTTCTGGGGCCGTT	114
Des (Desmin)	F: ATCCAGACCTTCTCTGCTCTCAA<br>R: TGTCTTTTTGGTATGGACTTCAGAAC	78
Vim (Vimentin)	F: TGGTTGACACCCACTCAAAAAG<br>R: TCTCATTGATCACCTGTCCATCTC	75
ACTA2 (α-SMA)	F: TTCCGCTGCCCAGAGACT<br>R: GATGCCCGCTGACTCCAT	60
36b4	F: CACTGGTCTAGGACCCGAGAAG<br>R: GGTGCCTCTGGAGATTTTCG	73
